# Evaluation of tumor-infiltrating lymphocytes (TILs) in molecular subtypes of an Indian cohort of breast cancer patients

**DOI:** 10.1186/s13000-022-01271-y

**Published:** 2022-11-21

**Authors:** Pooja M. Vaid, Anirudha K. Puntambekar, Nutan S. Jumle, Rituja A. Banale, Danish Ansari, Ruhi R. Reddy, Rohini R. Unde, Namrata P. Namewar, Devaki A. Kelkar, L. S. Shashidhara, Chaitanyanand B. Koppiker, Madhura D. Kulkarni

**Affiliations:** 1 Centre for Translational Cancer Research: a joint initiative of Indian Institute of Science Education and Research (IISER) Pune and Prashanti Cancer Care Mission (PCCM), Pune, India; 2 Department of Biological Sciences, Ashoka University, Sonipat, India; 3 Pathology Department, Ruby Hall Clinic, Pune, India; 4 Department of Pathology, Jehangir Hospital, Pune, India; 5 Prashanti Cancer Care Mission, Pune, Maharashtra India; 6 Indian Institute of Science Education and Research, Pune, India

**Keywords:** TILs, TNBC, Breast Cancer in India

## Abstract

**Objectives:**

Evaluation of tumor-infiltrating lymphocytes (TILs) distribution in an Indian cohort of breast cancer patients for its prognostic significance.

**Methods:**

A retrospective cohort of breast cancer patients from a single onco-surgeon’s breast cancer clinic with a uniform treatment strategy was evaluated for TILs. Tumor sections were H&E stained and scored for the spatial distribution and percent stromal TILs infiltration by a certified pathologist. The scores were analysed for association with treatment response and survival outcomes across molecular subtypes.

**Results:**

Total 229 breast cancer tumors were evaluated. Within spatial distribution categories, intra-tumoral TILs were observed to be associated with complete pathological response and lower recurrence frequency for the entire cohort. Subtype-wise analysis of stromal TILs (sTILs) re-enforced significantly higher infiltration in TNBC compared to HER2-positive and ER-positive tumors. A favourable association of higher stromal infiltration was observed with treatment response and disease outcomes, specifically in TNBC.

**Conclusion:**

Intra-tumoral TILs showed a higher proportion with favourable association with better patient outcomes in an Indian cohort, unlike western cohorts where both stromal and intra-tumoral TILs show similar association with prognosis. With further validation, TILs can be developed as a cost-effective surrogate marker for treatment response, especially in a low-resource setting such as India.

**Supplementary Information:**

The online version contains supplementary material available at 10.1186/s13000-022-01271-y.

## Key points

Key point 1:

The study is one of the first comprehensive evaluations of tumor-infiltrating lymphocytes in an Indian cohort of breast cancer patients.

Key point 2:

Intra-tumoral TILs presented in a higher proportion of patients, specifically in the TNBC subtype in an Indian cohort.

Key point 3:

Stromal TILs infiltration showed a higher distribution of TILs infiltration across TNBC tumors, where higher scores co-related with better therapy response and longer disease-free survival.

## Introduction

Breast Cancer is the leading cause of cancer-related female deaths in India, with close to a 50% mortality rate [1]. Though there are numerous targeted treatments are available for molecular subtypes with hormone receptor expression: ER, PR and/or HER2, as of now there is only targeted therapy available for TNBC which is directed towards immune response modulators [2]. Furthermore, TNBC is an aggressive subset of breast cancer with unpredictable response to therapy and hence higher rates of recurrence and lower overall survival [3, 4].

Worldwide, the prevalence of TNBC is 10–12% [5, 6], while in India, the prevalence of TNBC is reported to be significantly higher and up to 20–30% [7–9]. A greater proportion of TNBC cases in an Indian cohort are presented with aggressive clinicopathological features such as younger age, premenopausal status, and tumor with high grade [7]. With no targetable treatment and higher proportions of aggressive disease at incidence, TNBC poses a clinical management challenge in India, especially with a high proportion of incidences at a younger age.

The standard treatment for TNBC includes surgery for lymph node-negative patients and neoadjuvant chemotherapy (NACT) for patients with node metastasis, followed by surgery [10–12]. As of now, the treatment strategy of TNBC is determined by clinicopathologic features such as tumor size, proliferative index, lymph node involvement as well as a pathological response to chemotherapy in the neoadjuvant setting. TNBC is reported to show a better response to NACT compared to other subtypes, with 22 to 56% patients reported with pathologically complete response depending on the treatment regime [3, 13]. Pathological complete response in the TNBC subtype is shown to be associated with better disease-free survival [13]. Cases with the residual disease have been shown to have a significantly higher chance of recurrence within 1st three years of treatment and reduced overall survival [14, 15].

Recent studies have revealed tumor infiltrating lymphocytes (TILs) to be a promising predictive biomarker for therapy response, especially in TNBC [16–18]. Tumor infiltrating lymphocytes are cytotoxic lymphocytes infiltrating into the tumor and stromal regions as a host immune response [19, 20]. A greater extent of infiltration of lymphocytes, especially in the tumor stroma, enhances the anti-tumor effects of the therapy [21, 22]. Meta-analysis of 3770 patients with higher TILs scores associated with complete response to NACT in 50% of the TNBC patients, which was further associated with better long-term survival over three years, emphasizing the prognostic significance of TILs in TNBC [23, 24].

With a high incidence rate of TNBC in India, understanding TILs distribution with respect to treatment response and survival outcomes may help develop TILs as an accessible biomarker to predict treatment response in TNBC. TILs evaluation may provide a promise to predict a responsive subset of TNBC in India and help to de-escalate further chemotherapy.

Here we evaluate TILs with respect to clinicopathological features, treatment response and survival outcomes of a breast cancer patient cohort from a single surgeon and oncologist breast cancer unit in India [25].

## Materials and methods

### Patient tissue samples and meta-data

Primary breast tumor tissue (Formalin-fixed paraffin-embedded, FFPE) samples and associated de-identified patient metadata was received from the biobank [25], with appropriate patient consent and ethical approval (dated 21st July 2018 #IECHR/VB/2018/016). Patients who were diagnosed and underwent treatment from 2012 up to 15th July 2021 are included in the study cohort. Patient data, including diagnostic clinical and pathological reports, treatment regimens and post-treatment follow-up data up to last follow-up /recurrence date/death, was curated and digitized. All FFPE tissue blocks used were of primary (pre-treatment) tumor tissue. Of the 229 FFPE tumor samples, 179 were derived from true-cut core biopsy, and fifty were derived from naive tumor tissue excised during surgery.

Molecular subtypes of the breast tumors were determined based on immunohistochemistry and FISH reports from an accredited pathology lab. Samples were categorized into ER-positive, HER-positive, and TNBC subtypes based on ER/PR expression and HER2 scores. ER+ tumor samples were identified to be with more than 1% ER expression as positive for ER, HER2 IHC expression score of 0, 1+ or 2+ with FISH-negative report as negative for HER2 and positive or negative PR expression. Samples with HER2 IHC score of 3+ or score of 2+ with positive FISH and negative for ER expression with less than 1% expression, irrespective of PR expression were marked as HER2-positive subtype. Triple-negative samples were defined as the ones with less than 1% ER and less than 1% PR expression with HER2 IHC expression scores of 0, 1+ or 2+ and FISH-negative report.

NACT and ACT treatment was administered as per NCCN guidelines. The standard treatment for breast cancer patients with negative lymph nodes is surgery followed by ACT (adjuvant chemotherapy). For patients with lymph-node positivity, the standard treatment is NACT (neo-adjuvant chemotherapy), followed by surgery and ACT, and, if required, radiation therapy. TNBC patients (*n* = 26) were treated with Taxanes with or without Anthracycline/Cyclophosphamide (AC) or 5-Fluoro-Uracil/AC regimen as NACT and/or ACT regimen wherever appropriate. ER+ patients (*n* = 33) were treated with anti-estrogens such as Letrozole or Tamoxifen in case of NACT and AC + taxane regimen in case of ACT. For HER2 positive patients (*n* = 17), AC followed by Taxane with Trastuzumab was administered as NACT and Trastuzumab with or without a Taxane as NACT, and/or FAC and Taxane as ACT.

### Treatment response

Response to NACT was computed for 60 out of 76 patients who underwent NACT for whom clinical tumor size (cT) and node status (cN), as well as post-NACT tumor size (ypT) and node status (ypN), was available. Post-NACT, response to treatment was calculated by comparing cTcN values with ypTyPN. In case of residual DCIS or absence of residual tumor and absence of lymph node metastasis during pathological examination of surgically removed tissue, i.e., ypTisN0/ ypT0N0 status, the response is considered as pathological Complete Response (pCR). The presence of a residual tumor and/or lymph node metastasis in surgically removed tissue is referred to as Residual Disease (RD).

### Histopathology of FFPE tissue blocks

FFPE blocks of the primary tumor were processed for histopathology. Tumor sections of 4–5 μm were obtained using Leica Microtome RM2255. Tissue slides were deparaffinized. Each slide was cleaned and stained with a drop of undiluted Hematoxylin solution (Delafield, 38,803) in a humidifying chamber for 15 mins, followed by 1% eosin (Qualigen Q39312). The slides were then gradually dehydrated in ethanol solutions followed by Xylene. Slides were mounted in DPX (Q18404).

### Imaging of histopathology slides

All slides were imaged by OptraScan using the OS-15 bright field digital scanner at 400X magnification. Images were viewed using the ‘Image viewer’ software provided by Optra. The images were then converted to TIFF format and processed using Image Viewer Version 2.0.4 by OptraScan for scale bars.

### Spatial TILs scoring

Tumor-infiltrating lymphocytes (TILs) distribution in the tumor microenvironment was assessed from H&E sections. The spatial distribution of TILs was estimated based on the proximity of lymphocytes to the tumor cells, i.e. intra-tumoral TILs (I): TILs present within the tumor core and adjacent to tumor cells, peri-tumoral TILs (P): TILs present in the periphery of the tumor core, but restricted to surrounding stroma, stromal TILs (S): TILs present in the stromal tissue with no significant proximity to the tumor core, and desert TILs (D): TILs absent from both the tumor core as well as the stroma. For tumor samples with more than one type of spatial phenotype, a single score was taken based on the predominant presentation. Spatial distribution was scored twice independently with 100% concordance.

### Percent stromal tumor-infiltrating lymphocytes (sTILs) scoring

Percent TILs distribution of sTILs within stroma surrounding the tumor tissue was assessed from the H&E-stained histopathology section of primary tumor tissue. The scoring was done by the pathologist according to the recommendations of The TILs Working group [22]. The pathologist was blinded to the clinical data as well as molecular subtype information.

### Post-NACT sTILs scoring

For patients treated with NACT (*n* = 76), sTILs scores from post-NACT surgery samples were procured from the pathologist’s report. For patients with no report for sTILs scores, surgery H&E slides created at the time of original pathology were retrieved from storage and scored by the pathologists for sTILs.

### Response to treatment analysis

Response to Neo-adjuvant Chemotherapy (NACT) was calculated by comparing clinical tumor size (cT) and lymph node status (cN) to pathological tumor size (ypT) and pathological lymph node status (ypN). Post-NACT pathological status if yPT0 (no residual tumor) or ypTis (residual DCIS) and no lymph node involvement (ypN0), the response was defined as pathological Complete Response (pCR). For patients with residual tumor (ypT1-ypT4) and/or lymph node metastasis (ypN1–3) in a post-NACT setting, the response was noted as Residual Disease (RD).

Response to NACT across the subtypes is then analyzed using a 3 × 4 Chi-square contingency test. Box plot for percent sTILs scores according to the response to NACT, i.e., pCR and RD, is plotted using GraphPad Prism v.5. Mean sTILs with S.E. according to the response to NACT for each subtype is computed and plotted by using GraphPad Prism v.5.

sTILs scores of primary biopsy and post-treatment surgery tissues were plotted using a before-after graph using GraphPad Prism v.5. Paired t-test was performed to assess the significance of the difference in mean sTILs scores between primary and post-NACT tumor tissue.

### Statistical analysis

All statistical analysis was done using GraphPad Prism v.5. A demographic table was prepared using IBM SPSS Statistics v. 21.0.0.0. The distribution of clinicopathological characteristics within the cohort and breast cancer subtypes was analyzed using a 2 × 3 (4 × 3 in case of tumor size) Chi-square contingency test. Mean age across subtypes was compared using the Mann Whitney test. Column statistics for sTILs were computed on GraphPad Prism v.5 to calculate mean and standard error across each sub-category of clinical characteristics. A significant difference in distribution across ER+, HER2+ and TNBC sTILs scores for clinicopathological characteristics were analyzed with the Kruskal-Wallis test. Individual comparison across two groups was analyzed by the Mann-Whitney test. All graphs were plotted using GraphPad Prism v.5.

### Survival outcome analysis

Survival outcomes were computed as follows: disease-free survival (DFS) was calculated as time in months from the date of surgery till the date of recurrence or last follow-up date. Overall survival (OS) was calculated as time in months from the diagnosis date (biopsy date) till the last follow-up date or date of death due to disease. Kaplan-Meier survival plots for DFS and OS for up to 5-years follow-up time were plotted, and survival probabilities were computed by Log-rank, Breslow, and Tarone-Ware cores towards 5-years DFS and OS using IBM SPSS Statistics v 21.0.0.0.

## Results

### Breast cancer cohort characteristics

Primary tumor tissue samples of IDC patients were identified and retrieved for 229 cases from the breast cancer biobank. Demographic and clinical characteristics of the cohort are presented according to their molecular subtypes (ER+, HER2+ and TNBC) in Table 1. The average age of the cohort of 229 breast cancer patients is 54.4, ranging from 28 to 86 years. TNBC patients (*n* = 81) presented with a significantly higher proportion of younger age (mean age of 51.8 ± 12 years) and premenopausal patients (39.4%) compared to ER+ and HER2+ patients (mean age of 56.1 ± 12 and 55.1 ± 12 years; respectively) with 28.0 and 21.6% premenopausal cases. TNBC reflected a significantly higher proportion of high-grade tumors (76.5% grade III) compared to other subtypes (25.6 and 56.9%, respectively). Clinical and pathological tumor size, lymph node positivity, stage and LVI did not differ significantly across all three subtypes. Overall and disease-free survival data were available for 219 and 197 patients, respectively. The cohort had an average follow-up of 22 months and a median follow-up of 14 months.

**Table 1:** Demographic table of the breast cancer cohort, with clinical characteristics across subtypes

No. of patients		All BC	ER+	HER2+	TNBC	*p*-values
		229	90	58	81	
**Age (*n*** **= 227)**	**(Mean ± S.D)**	54.4 ± 12.2	56.1 ± 12.5	55.1 ± 11.9	51.8 ± 11.9	0.0635*
	**Early (> 50)**	86 (37.9%)	29 (32.2%)	21 (36.2%)	36 (45.6%)	0.1941
	**Late (≤ 50)**	141 (62.1%)	61 (67.8%)	37 (63.8%)	43 (54.4%)	
	**NA**	2	0	0	2	
**Menopausal status (*n*** **= 192)**	**pre**	58 (30.2%)	21 (28.0%)	11 (21.6%)	26 (39.4%)	0.0992
	**post**	134 (69.8%)	54 (72.0%)	40 (78.4%)	40 (60.6%)	
	**NA**	37	15	7	15	
**Grade (*n*** **= 229)**	**Low (I/II)**	111 (48.5%)	67 (74.4.%)	25 (43.1%)	19 (23.5%)	**< 0.0001**
	**High (III)**	118 (51.5%)	23 (25.6%)	33 (56.9%)	62 (76.5%)	
	**NA**	0	0	0	0	
**Tumor size (cT) (*n*** **= 216)**	**T1**	66 (30.6%)	35 (40.2%)	13 (24.1%)	18 (24%)	0.2913
	**T2**	136 (63%)	47 (54.0%)	38 (70.4%)	51 (68%)	
	**T3**	12 (5.5%)	4 (4.6%)	3 (5.6%)	5 (6.7%)	
	**T4**	2 (0.9%)	1 (1.1%)	0 (0.0%)	1 (1.3%)	
	**NA**	13	3	4	6	
**LN status (cN) (*n*** **= 206)**	**negative**	65 (31.6%)	31 (36.9%)	14 (28.0%)	20 (27.8%)	0.3904
	**positive**	141 (68.4%)	53 (63.1%)	36 (72.0%)	52 (72.2%)	
	**NA**	23	6	8	9	
**Clinical_Stage (*n*** **= 209)**	**Early(<IIB)**	84 (40.2%)	41 (47.7%)	15 (30%)	28 (38.4%)	0.1185
	**Late(≥IIB)**	125 (59.8%)	45 (52.3%)	35 (70%)	45 (61.6%)	
	**NA**	20	4	8	8	
**pT (primary tissue, no NACT) (*n*** **= 128)**	**T0**	6 (4.7%)	4 (8.7%)	0 (0.0%)	2 (4.4%)	0.4153
	**T1**	30 (23.5%)	14 (30.5%)	5 (13.5%)	11 (24.4%)	
	**T2**	85 (66.4%)	26 (56.5%)	30 (81.1%)	29 (64.4%)	
	**T3**	4 (3.1%)	1 (2.2%)	1 (2.7%)	2 (4.4%)	
	**T4**	3 (2.3%)	1 (2.2%)	1 (2.7%)	1 (2.2%)	
	**NA/NACT_Yes**	101	44	21	36	
**pN (primary tissue, no NACT) (*n* = 128)**	**negative**	86 (67.2%)	31 (67.4%)	22 (59.5%)	33 (73.3%)	0.4119
	**positive**	42 (32.8%)	15 (32.6%)	15 (40.5%)	12 (26.7%)	
	**NA/NACT_Yes**	101	44	21	36	
**pathological Stage (primary tissue, no NACT) (*n* = 128)**	**Early(<IIB)**	85 (66.4%)	31 (67.4%)	21 (56.8%)	33 (73.3%)	0.2819
	**Late(≥IIB)**	43 (33.6%)	15 (32.6%)	16 (43.2%)	12 (26.7%)	
	**NA/NACT_Yes**	101	44	21	36	
**LVI (*n* = 229)**	**negative**	184 (80.3%)	67 (74.4%)	47 (81.0%)	70 (86.4%)	0.1426
	**positive**	45 (19.7%)	23 (25.6%)	11 (19.0%)	11 (13.6%)	
	**NA**	0	0	0	0	
**NACT (*n*** **= 218)**	**No**	142 (65.1%)	52 (62.2%)	40 (70.2%)	50 (65.8%)	
	**Yes**	76 (34.9%)	33 (38.8%)	17 (29.8%)	26 (34.2%)	
	**NA**	11	5	1	5	
**PCR status after NACT (***n*** = 60)**	**pCR**	15 (25%)	3 (11.1%)	2 (22.2%)	10 (41.7%)	**0.0414**
	**RD**	45 (75%)	24 (88.9%)	7 (77.8%)	14 (58.3%)	
	**NA**	17	7	8	2	
**Survival outcomes**	**No. followed-up**	219	87	54	78	
	**Time in Months Mean (range)**	22(0–172)	18(0–84)	24(0–172)	24(0–132)	
	**Median months**	14	12	14	18	
	**# Recurred (local, distant)**	18	5	3	10	
	**# Death due to disease**	6	2	0	4	

A cohort of IDC patients was grouped according to the ER+, HER2+ and TNBC subtypes. Clinical parameters such as age at diagnosis, menopausal status, tumor grade, radiological and pathological tumor size, lymph node positivity, stage, and LVI are listed. The number of patients is listed according to the clinical variables reported at the time of diagnosis. For patients who did not receive NACT/NAHT, pT and pN retrieved from the surgery pathology report are noted. For patients who received NACT/NAHT, pathological response to the therapy is noted based on their ypTypN status. A total number of patients with follow-up, mean time to follow-up and follow-up status are also noted. The distribution of clinical parameters across the ER+, HER2+ and TNBC subtypes, was analyzed using the 2*3 (4*3 and 5*3 in case of clinical and pathological tumor size) χ2 contingency test with GraphPad Prism v.5. Bold font indicates significant p-values

*For comparing mean age differences among the subtypes, one way ANOVA was performed

*LVI* lymphovascular invasion, *NACT* Neoadjuvant chemotherapy, *pCR* pathological complete response

Out of 229 patients, 76 (34.9%) received neoadjuvant chemotherapy (NACT) according to their clinical and hormone receptor expression status as described in the methods section. Of these 76, 15 patients showed a complete pathological response (pCR) as assessed by ypT0 (or Tis) ypN0 status. The number of patients with pCR and residual disease (RD) across all three subtypes showed significantly different distribution (*p*-value = 0.04) where the highest pCR rates (41.7%; 10 out of 24) were observed for TNBC patients.

### TILs distribution across molecular subtypes

**Fig. 1:**
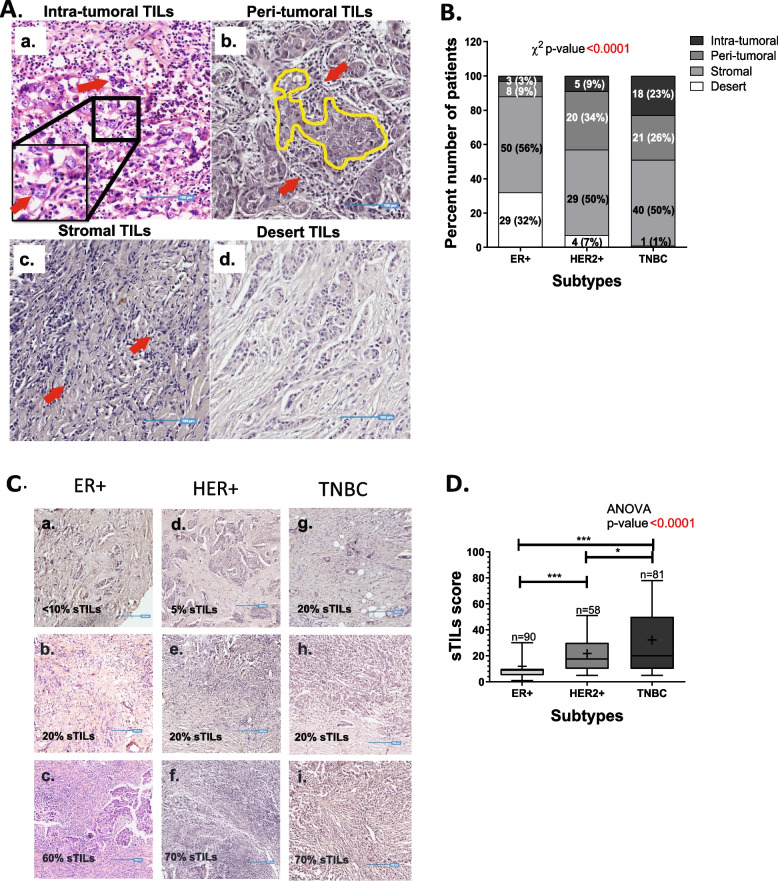
TILs spatial phenotype and stromal TILs (sTILs) scores across the three subtypes of breast cancer. **A**. Representative images depicting the spatial distribution of TILs in breast cancer. Representative images of four phenotypes of spatial TILs are presented here for a. Intra-tumoral TILs, b. Peri-tumoral TILs, c. Stromal TILs and d. Desert TILs. The yellow area represents the tumor area. Red arrows indicate TILs. Blue lines are the scale bars representing 100 μm. **B**. Stacked bar graph representing percent number of patients across the subtypes for four phenotypes of spatial TILs. The number of patients and percentage is shown as n (%). Distribution of number of patients across the spatial phenotypes according to their subtypes is tested by 3*4 χ2 (Chi-Square) contingency test. **C**. Representative images depicting stromal TILs distribution. Representative images of sTILs scores are presented here for ER+ (left panel), HER2+ (middle panel) & TNBC (right panel) with sTILs scores mentioned at the left bottom. Blue lines are the scale bars representing 200 μm. **D**. Box plot shows the distribution of sTILs scores across ER+, HER2+ and TNBC subtypes. The horizontal line represents the median. Error bars represent 10th and 90th percentile values. The number of patient samples (n) are shown in the box plot. The distribution of the sTILs scores amongst subtypes was analyzed for statistical significance with the Kruskal Wallis test and individual comparison between two subtypes by Mann-Whitney test, using GraphPad Prism v.5. *represent *p*-value of < 0.05, *** represents p-value < 0.0005

Mononuclear lymphocytes were scored for each tumor tissue based on H&E staining. The spatial distribution of TILs was classified into four phenotypes based on the proximity of lymphocytes to the tumor cells. Intra-tumoral: lymphocytes infiltrated within the tumor core and in close proximity to tumor cells, peri-tumoral: lymphocytes close to tumor core, but restricted to surrounding stroma, stromal: lymphocytes restricted to stromal tissue and distant from the tumor core and desert: where lymphocytes were absent in tumor core as well as stromal tissue. Representative images of each phenotype are shown in Fig. 1A. Distribution analysis for spatial phenotypes across molecular subtypes revealed TNBC with a higher proportion of intra-tumoral TILs (23%) with hardly 1% patients with desert TILs phenotype. While ER+ patients reflected a high proportion of desert TILs phenotype (32%) and 3% of tumors with intra-tumoral TILs (Fig. 1B). More than 50% of IDC tumors harboured stromal TILs across all subtypes i.e., 56% (*n* = 50) for ER+ patients, 50% for both HER2+ and TNBC patients (*n* = 29 and *n* = 40; respectively). Peri-tumoral TILs were seen in a higher proportion in HER2+ patients compared to other subtypes (34%, *n* = 20) (Fig. 1B).

Stromal TILs were quantified by a certified pathologist since more than 50% of the tumors were presented with stromal infiltration of lymphocytes. Percent Stromal TILs (sTILs) infiltration was evaluated as sTILs score for each tumor tissue, and sTILs scores were then compared across the three molecular subtypes (Fig. 1C). TNBC tumors harbored a wider range of sTILs scores (1–90%) as compared to the other subtypes (Fig. 1D). Mean sTILs score was significantly higher in TNBC (32.2 ± 2.8, *n* = 81) compared to ER+ and HER2+ tumors (11.9 ± 1.4, *n* = 90 and 21.7 ± 2.4, *n* = 58, respectively).

### Stromal TILs percentage and association with clinical parameters

The distribution of percent sTILs scores across clinical features of the cohort is presented in Table S1. Average sTILs scores showed even distribution across age, menopausal status, and lymph node status. Tumor grade showed significantly higher sTILs percentages in high-grade tumors (32.1 ± 2.2) vs low-grade tumors (10.4 ± 0.9). Tumors with smaller sizes showed higher sTILs compared to larger tumors, for both clinical - cT as well as pathological - pT size.

sTILs scores were correlated across molecular subtypes and their clinical parameters (Table 2). Mean sTILs score was significantly higher in TNBC across all the clinical parameters compared to ER+ and HER2+ patients except for lymph node involvement and lymph vascular invasion (LVI). For lymph node involvement and LVI, ER+ and HER2+ but not TNBC tumors show increased infiltration in node-positive tumors in contrast to previously published meta-analysis [24] for both clinical and pathological reports.

**Table 2:** Mean sTILs scores with respect to clinicopathological features of breast cancer subtypes

TILs distribution against subtypes w.r.t clinical parameters		ER+ (*n* = 90)	HER2+ (*n* = 58)	TNBC (*n* = 81)	***p-values (Kruskal-Wallis test)***
**No. Of patients (*n* = 229)**	**(Mean ± S.E)**	11.9 ± 1.4 (*n* = 90)	21.7 ± 2.4 (*n* = 58)	32.2 ± 2.8 (*n* = 81)	**< 0.0001**
**Age, (*n*** **= 227)**	**Early (< 50)**	14.9 ± 3.5 (*n* = 29)	18.1 ± 3.1 (*n* = 21)	34.3 ± 4.1 (*n* = 36)	**< 0.0001**
	**Late (≥ 50)**	10.5 ± 1.3 (*n* = 61)	23.8 ± 3.4 (*n* = 37)	30.8 ± 3.9 (*n* = 43)	
	**NA**	0	0	2	
**Menopausal status, (*n* = 192)**	**Pre**	16.9 ± 4.7 (*n* = 21)	12.7 ± 2.6 (*n* = 11)	31.7 ± 4.9 (*n* = 26)	**< 0.0001**
	**Post**	10.3 ± 1.5 (*n* = 54)	24.4 ± 3.3 (*n* = 40)	33.8 ± 4.3 (*n* = 40)	
	**NA**	15	7	15	
**Grade, (*n* = 229)**	**I/II**	7.9 ± 0.8 (*n* = 67)	12.0 ± 1.8 (*n* = 25)	17.1 ± 3.5 (*n* = 19)	**< 0.0001**
	**III**	23.7 ± 4.4 (*n* = 23)	29.1 ± 3.6 (*n* = 33)	36.8 ± 3.3 (*n* = 62)	
	**NA**	0	0	0	
**Tumor size (cT), (*n* = 216)**	**T1, T2**	12.2 ± 1.5 (*n* = 82)	22.1 ± 2.7 (*n* = 51)	32.7 ± 3.1 (*n* = 69)	**< 0.0001**
	**T3, T4**	11.8 ± 4.7 (*n* = 5)	18.3 ± 7.3 (*n* = 3)	22.5 ± 6.6 (*n* = 6)	
	**NA**	6	8	9	
**LN status (cN), (*n* = 206)**	**Negative**	10.5 ± 2.2 (*n* = 31)	16.4 ± 4.1 (*n* = 14)	34.4 ± 5.9 (*n* = 20)	**< 0.0001**
	**Positive**	13.3 ± 2.1 (*n* = 53)	25.0 ± 3.4 (*n* = 36)	29.6 ± 3.4 (*n* = 52)	
	**NA**	6	8	9	
**Clinical Stage, (*n* = 209)**	**Early (<IIB)**	9.7 ± 1.7 (*n* = 41)	16.3 ± 3.8 (*n* = 15)	37.1 ± 5.3 (*n* = 28)	**< 0.0001**
	**Late (≥IIB)**	14.4 ± 2.4 (*n* = 45)	25.5 ± 3.5 (*n* = 35)	28.9 ± 3.5 (*n* = 45)	
	**NA**	4	8	8	
**LVI, (*n* = 229)**	**Negative**	10.8 ± 1.3 (*n* = 67)	20.9 ± 2.7 (*n* = 47)	32.7 ± 3.0 (*n* = 70)	**< 0.0001**
	**Positive**	15.3 ± 4.3 (*n* = 23)	25.0 ± 6.1 (*n* = 11)	28.7 ± 8.1 (*n* = 11)	
	**NA**	0	0	0	
**Tumor size (pT), (*n* = 128) NACT_No**	**T0, Tis**	23.8 ± 12.5 (*n* = 4)	0	50.0 ± 30.0 (*n* = 2)	**< 0.0001**
	**T1, T2**	10.2 ± 1.7 (*n* = 40)	19.1 ± 3.0 (*n* = 35)	33.3 ± 4.2 (*n* = 40)	
	**T3, T4**	17.5 ± 12.5 (*n* = 2)	20.0 ± 10.0 (*n* = 2)	20.0 ± 10.0 (*n* = 3)	
	**NA/NACT_Yes**	44	21	36	
**LN status (pN), (*n* = 128) NACT_No**	**Negative**	10.5 ± 2.2 (*n* = 31)	17.7 ± 3.5 (*n* = 22)	34.8 ± 4.7 (*n* = 33)	**< 0.0001**
	**Positive**	14.1 ± 3.8 (*n* = 15)	21.3 ± 5.0 (*n* = 15)	28.8 ± 7.3 (*n* = 12)	
	**NA/NACT_Yes**	44	21	36	
**Pathological Stage, (*n* = 128)** **NACT_No**	**Early (<IIB)**	10.5 ± 2.2 (*n* = 31)	18.1 ± 3.7 (*n* = 22)	35.4 ± 4.6 (*n* = 33)	**< 0.0001**
	**Late (≥IIB)**	14.1 ± 3.8 (*n* = 15)	20.6 ± 4.8 (*n* = 16)	27.1 ± 7.4 (*n* = 12)	
	**NA/NACT_Yes**	44	21	36	

Mean ± S.E sTILs scores according to the molecular subtypes are presented across clinicopathological parameters including age at diagnosis, menopausal status of patients, tumor grade, radiological and pathological tumor size, lymph node status and stage, and LVI

The statistical analysis was performed using GraphPad Prism v.5. Kruskal Wallis test was performed to compute the significance for mean sTILs scores across breast cancer subtypes. The bold font indicates significant p-values. *LVI- lymphovascular invasion

### TILs distribution and its correlation with response to NACT

Out of the 60 patients that received NACT, a significantly higher proportion of TNBC patients showed pCR (42%) compared to ER+ (11%) and HER2+ (22%) patients (Table 1). To determine the role of TILs in NACT response, an association between the spatial phenotype of TILs as well percent stromal infiltration scores were evaluated.

**Fig. 2:**
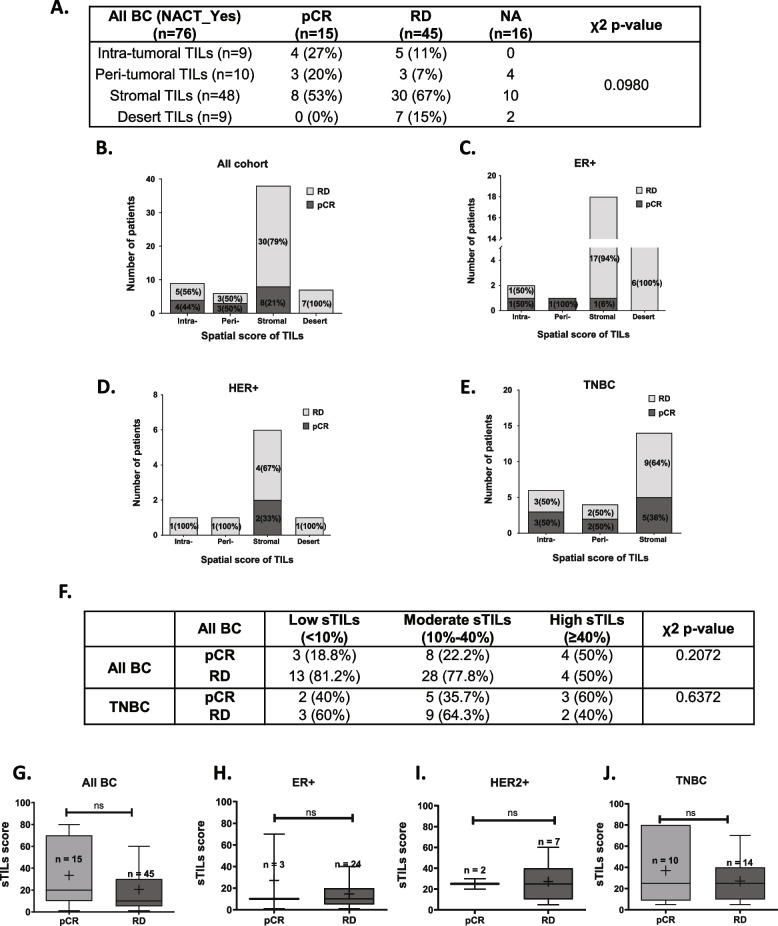
Spatial TILs phenotype and sTILs scores association with NACT response. **A** Spatial TILs phenotype and its association with response to NACT. Table showing the number of patients according to their TILs spatial phenotype and pathological response, where the response is measured as pCR and RD. Distribution of the number of patients across four phenotypes of spatial TILs was analyzed with the 4*2 χ2 contingency test using GraphPad Prism v.5. The bold font indicates significant *p*-values. **B-E** Spatial TILs phenotypes and its association with response to NACT across subtypes. Stacked bar graph representing percent number of patients with each spatial TILs phenotype with respect to NACT response. The therapy response is reported as pCR and RD according to the spatial TILs phenotype of the tumor for B. the IDC cohort, C. ER+ subtype D. HER2+ subtype E. TNBC subtype. The number of patients and percentage is shown in each bar as n (%). **F** Table showing the distribution of IDC and TNBC patients with pCR or RD with respect to binned sTILs score; Low sTILs (< 10%), Moderate sTILs (10–40%) and High sTILs (≥40%). χ2 p-value was computed using GraphPad Prism V.5. G-J; Box plots depicting mean sTILs scores separated according to the response to NACT for the cohort and the three subtypes. The number of tissue samples (n) is shown on top of each bar. Error bars represent 10th and 90th percentile values. Mann Whitney test was performed to analyze significant distribution of mean sTILs scores. *p*-value < 0.05 is represented with ‘*’, < 0.01 with ‘**’ and, < 0.0001 with ‘***’. ns = non-significant. GraphPad Prism v.5 was used for the graphs and statistical calculations

Patients with pathological complete response showed a higher proportion of intra-tumoral and peri-tumoral TILs phenotypes (27 and 20%, respectively) compared to patients with residual disease (11 and 7%, respectively) (Fig. 2A, B). Tumors (*n* = 7) with desert TILs phenotype showed RD, where 100% of the patients were left with residual disease (RD) post-NACT (Fig. 2A, B).

Analysis of spatial phenotype across subtypes shows the highest proportion of ER+ tumors harboring stromal and desert phenotype where patients are left with residual tumor (RD) post-NACT (Fig. 2C), while a small proportion of tumors with stromal TILs had pCR for HER2+ subtype (Fig. 2D). For TNBC tumors, pCR was observed for all spatial phenotypes with a higher percentage of response for intra-tumoral and per-tumoral phenotypes (Fig. 2E).

Next, sTILs scores binned into three categories – low (< 10%), moderate (10–40%) and high (> 40%) according to the TILs working group guidelines [22] were analyzed for its association with response to NACT (Fig. 2F). The highest proportion of patients (50%) with pCR had high sTILs compared to patients with RD, where 81.2% of patients had low sTILs scores. A similar trend was seen for TNBC patients, where 60% of patients with high sTILs score showed pCR.

Patients with complete response showed a wider range of percent sTILs scores compared to the ones who had residual disease (RD) post-NACT (Fig. 2G). TNBC patients with pCR showed a similar trend of higher and wider sTILs scores compared to patients with RD (Fig. 2J), while the opposite trend was observed for ER+ and HER2+ patients (Fig. 2H, I).

### sTILs scores comparison between pre- and post-NACT

**Fig. 3:**
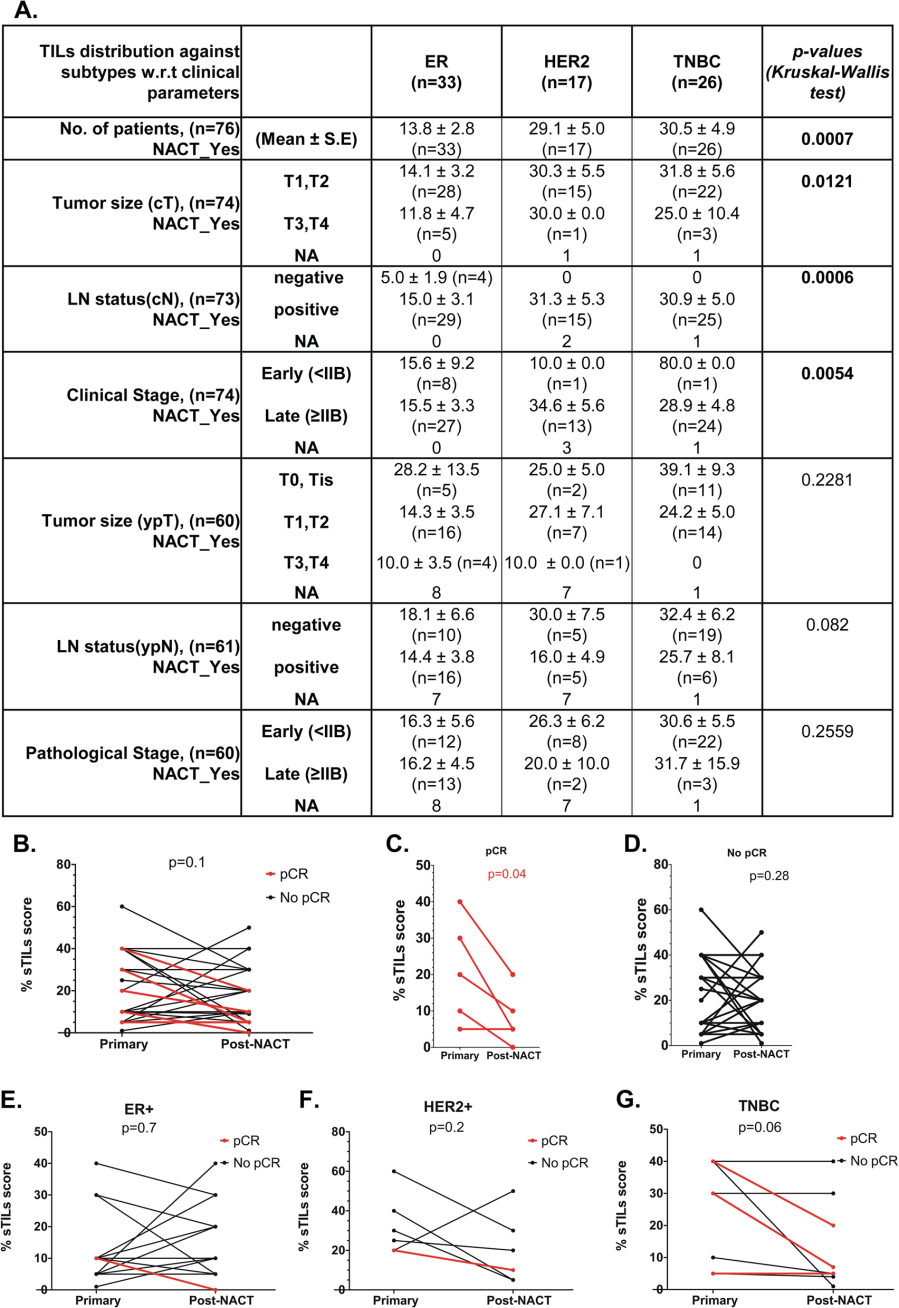
sTILs scores compared between pre-NACT and post-NACT tumor tissue. **A** Table represents mean ± S.E sTILs scores across clinicopathological parameters, including radiological and pathological tumor size, lymph node status for NACT-treated patients, according to the molecular subtypes. The statistical analysis was done using GraphPad Prism v.5. Kruskal Wallis test was performed to compute the significant difference in mean sTILs scores across breast cancer subtypes. The bold font indicates signifiant *p*-values. **B-G** Before-after graph depicting sTILs scores for pre- and post-NACT tumor tissue. Individual sTILs scores for each paired sample is shown for patients who received NACT. B; the IDC cohort, C; patients with pCR and D; patients with RD, E; the IDC cohort, E; ER+ subtype, F; HER2+ subtype and G, TNBC subtype. Paired t-test was performed to test the difference in mean between sTILs scores of primary and post-NACT tissue. The red lines indicate patients who showed pathological complete response (pCR), and black lines indicate patients who had the residual disease (RD). The bold font indicates a significant *p*-value. *p*-value < 0.05 is represented with ‘*’, < 0.01 with ‘**’ and, < 0.0001 with ‘***’. ns = non-significant. GraphPad Prism v.5 was used for the graphs and statistical calculations

To evaluate if there is an association between treatment response and changes in stromal infiltration through the treatment, sTILs scores were assessed for any alteration from pre-NACT to a post-NACT setting. The change in the scores between pre- and post-NACT settings are plotted according to the treatment response (Fig. 3B). Patients with pCR (*n* = 4) showed a significant decrease in sTILs scores from pre-NACT to post-NACT setting (Fig. 3C), while patients with residual disease (RD) showed no significant change (Fig. 3D). When compared across subtypes, no significant correlation was observed for ER+ and HER2+ tumors (Fig. 3E, F), while TNBC tumors showed a significant association of reduced stromal infiltration from pre-treatment to post-treatment setting with pCR but not with RD (Fig. 3G).

### TILs distribution and its association with survival outcomes

**Fig. 4:**
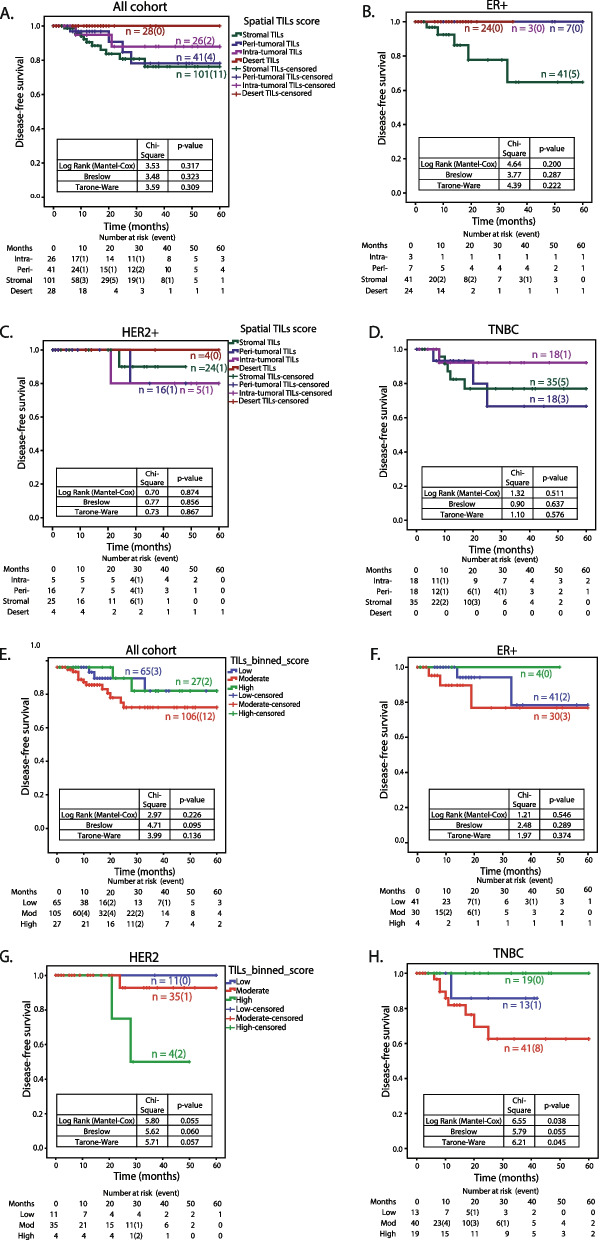
Disease-free survival (DFS) for five-year follow-up according to the spatial TILs phenotype and sTILs scores. Disease-free survival (DFS) was calculated as number of months from the date of surgery till the recurrence diagnosis date or last follow-up date up to five years. Kaplan-Meier survival plots for disease-free survival (DFS) are plotted. Each drop shown as a vertical line represents an event i.e., local, or distant recurrence. Survival probability with respect to the spatial TILs phenotype is analyzed using IBM SPSS Statistics v. 21.0.0.0. The number of patients at risk at each time interval of 10 months from 0 to 60 months is shown. The number of events is indicated in brackets at respective time points. **A-D** DFS for the four phenotypes of the spatial TILs; Intra-tumoral TILs, Peri-tumoral TILs, Stromal TILs, and Desert TILs for A; the IDC cohort, B; ER+ subtype C; HER2+ subtype and D; TNBC subtype. In the graph, X-axis represents the time scale in months, and Y-axis represents the survival probability. The green line indicates patients with stromal TILs, the blue line indicates patients with peri-tumoral TILs, the purple line indicates patients with intra-tumoral TILs, and the red line indicates patients with desert TILs phenotype. **E-H** DFS with respect to binned percent stromal TILs infiltration score. Kaplan-Meier survival plots for disease-free survival (DFS) according to low, moderate & high sTILs score bins for E; the IDC cohort, F; ER+ subtype, G; HER2+ subtype and H; TNBC. In the graph, X-axis represents the time scale in months, and Y-axis represents the survival probability. The blue line indicates patients with low sTILs scores, and the red line indicates patients with moderate scores & green indicates high sTILs scores

Spatial TILs phenotypes (stromal, peri-, intra- and desert) were analyzed for their association with survival outcomes, disease-free survival (DFS) and overall survival (OS) (Fig. 4 and S3). Surprisingly, for the entire cohort of IDC patients, desert phenotype showed longer event-free survival (DFS), followed by intra-tumoral phenotype, while stromal and peri-tumoral TILs phenotypes showed shorter survival, though not significant (Fig. 4A). ER+ subtype showed worst disease-free survival for patients with stromal TILs phenotype (Fig. 4B), while no specific association was observed for HER2+ subtype. For TNBC patients, this cohort showed better survival for patients with intra-tumoral lymphocyte infiltration over stromal or peri-tumoral (Fig. 4D). For overall survival across the cohort and subtypes, again, no specific association was observed for spatial TILs phenotypes (Fig. S3).

Further, following TILs working group guidelines, percent stromal TILs scores were binned into three categories: low (< 10%), moderate (10–40%) and high (> 40%) and were analyzed for association with the survival outcomes (Fig. 4 and S3). The cohort showed no specific association of binned sTILs scores with disease-free survival (Fig. 4E). Subtype wise analysis with respect to binned sTILs scores showed high sTILs scores in TNBC tumors to be significantly associated with longer disease-free survival as compared to low and moderate sTILs scores (Fig. 4H). ER+ patients showed a similar trend as that of TNBC, though ER+ subtypes had very few patients with high sTILs scores (Fig. 4F). In contrast, high sTILs showed poor survival in HER2+ patients (Fig. 4G). For overall survival, the cohort comprised of 6 events (death due to disease), where 5 out of 6 harbored moderate sTILs scores, while patients with high sTILs did not have any events within the follow-up period (Fig. S3 D-F).

## Discussion

Infiltrating tumor lymphocytes are being evaluated in clinical trials as a surrogate marker for treatment response in breast cancer, as summarized recently by TILs working group [26] and specifically in TNBC [27]. TILs scores serve as a potential prognostic marker, not only as a surrogate marker to de-escalate toxic and expensive chemotherapy, but also due to its cost-effectiveness as a diagnostic tool, especially in low-resource countries such as India. With this anticipation, this study presents one of the first TILs evaluations in an Indian cohort of breast cancer patients. Detailed evaluation of lymphocytes’ spatial distribution and stromal infiltration scores for their association with treatment response and patient outcomes is presented here.

The cohort of 229 breast cancer with 81 TNBC, 90 ER+ and 58 HER2+ patients reflected the uniform distribution of clinicopathological parameters except for age and grade. TNBC patients presented with younger mean age as compared to other subtypes and comprised of a higher proportion (45%) of young age (< 50 years) and (39%) premenopausal patients. Younger age at incidence for TNBC subtype in high frequency is reported earlier in two meta-analyses for breast cancer patients in India [7, 9]. The cohort shows a similar and significant trend of age distribution across breast cancer patients. This is in contrast to the western cohorts where young age TNBCs present at 29–34% [28, 29].

TILs in the tumor microenvironment influence the overall breast cancer prognosis and response to treatment [26]. CD8+ T-cell population has been used to define spatial phenotypes in breast cancer based on the infiltration in the tumor core and/or stroma [30]. A higher proportion of cytotoxic T cells in the tumor core has been shown to be associated with a better prognosis. Therefore, the spatial context of TILs with respect to the tumor was analyzed for the Indian cohort for its association with response to NACT and survival outcomes. More than 50% of patients in each subtype presented with stromal TILs, while 11.3% patients harbored intra-tumoral TILs (*n* = 26), where intra-tumoral TILs associated with better outcomes in IDC patients. Specifically in TNBC subtypes, higher proportion of tumors (23%) harbored intra-tumoral TILs, unlike the western cohort, where iTILs were observed in a small proportion of IDC as well as TNBC patients [22]. In BIG 02–98 trial data, median intra-tumoral infiltration: iTILs score of 2%, while median stromal TILs score was 10% for a cohort size of 2000 patients was observed [31]. With a higher number of patients with intra-tumoral TILs, specifically in the TNBC subset of the cohort, their prognostic role needs to be further evaluated within Indian cohorts.

Two comprehensive meta-analyses for stromal TILs association with patient outcomes for large cohorts of breast cancer patients have been studied earlier [23, 24]. Denkert et al. in 2018, where pooled data from 6 clinical trials with 3771 patients where sTILs scores are binned as 60% and above as the cut-off for high sTILs subgroup [23]. In another meta-analysis by Loi et al., sTILs scores for 2148 TNBC patients were analyzed with high sTILs cut-off as 30% and above [24]. Despite different cut-off for high sTILs at 40%, subtype wise comparison of sTILs scores from our cohort co-related well with reported studies [22, 23], where TNBC subtype presented with higher mean sTILs scores compared to that of ER+ and HER2+ subtypes.

Stromal TILs scores in the cohort were uniformly distributed irrespective of the clinic-pathological parameters of the tumors, except for grade and tumor size. Higher sTILs were seen in grade 3 TNBC tumors. Similar association was seen by Loi et al., where a higher sTILs score was significantly associated with high-grade tumors [24]. Within TNBC, higher sTILs scores co-related with better disease-free outcomes, as reported earlier by Denkert and colleagues [23].

For patients who received NACT (*n* = 60), 42% of TNBC patients showed complete pathological response post-NACT as opposed to 11 and 22% of ER+ and HER2+ subtypes, respectively. This is in line with the literature, where the TNBC subtype has been reported to have a better response to therapy [3, 13, 32]. Even with the limited number of patients that received NACT, higher sTILs scores were associated with complete pathological response. Partial or lack of response to therapy co-related with a lower sTILs scores specifically for the TNBC subtype, similar to that has been reported in western population [23]. The trends observed in our cohort analysis are in line with the established association of sTILs distribution and TNBC outcomes.

In this study, changes in stromal infiltration of lymphocytes in tumor tissue from pre- to post-treatment settings were assessed between paired samples. Interestingly, a significant decrease in the lymphocyte infiltration was observed for patients who showed complete pathological response post-NACT. There is another study where a cohort of 104 TNBC patients [33] showed that increase in sTILs in post-NACT samples associated with better disease-free survival compared to patients where a decrease in sTILs was observed. With such contrasting observations within small sets of cohorts, whether such changes in sTILs in a post-NACT setting can be directly implicated in complete response and better outcomes needs to be evaluated further.

This is the first time an Indian cohort of breast cancer patients is evaluated to assess whether the predictive benefit of TILs can be extended towards breast cancer patients in India, especially when Indian cohorts show significantly different demographic distribution. Our analysis reflects similar trends for TILs association with clinical parameters and patient outcomes despite the demographic differences. Though, finer differences are revealed in this analysis, such as a higher proportion of patients with intra-tumoral TILs with better outcomes over stromal TILs. Thus, extending the utility of the TILs as a putative predictive marker for treatment response for TNBC in India will require further validation with a larger cohort.

TILs assessment can be done using inexpensive and traditional histopathology methods, routinely used even in low-resource countries like India. TILs assessment from histopathology images of tumor slides can be developed as a robust digital pathology tool that can be incorporated for treatment utility predictions and can be availed across the country.

With further validation in a larger cohort across India, TILs have the potential to be a predictive biomarker for chemotherapy response. The newer treatments, like immune checkpoint inhibitor therapies that depend on PD1/PDL1 diagnostic markers, have been shown to use TILs as an associative marker for PD1/PDL1 expression in recent trials such as KEYNOTE-086 [34] and IMPASSION130 [35]. PD1/PDL1 diagnosis is expensive in India and hence with very low penetrance of utility due to low-recourse settings and a high proportion of low-income communities. If TILs is approved to be a surrogate marker for PD1/PDL1 expression, it will serve as a cost-effective tool for treatment management decisions.

## Supplementary Information

**Additional file 1.**
