# Supplementary material for: Evaluation of tumor-infiltrating lymphocytes (TILs) in molecular subtypes of an Indian cohort of breast cancer patients
Source: Diagn Pathol. 2022 Nov 21;17:91. doi: 10.1186/s13000-022-01271-y (PMC9677664; doi:10.1186/s13000-022-01271-y)
Supplement: Supplementary file 1 — Additional file 1. [file 13000_2022_1271_MOESM1_ESM.docx]

**Table S1: Mean sTILs scores with respect to clinicopathological features of the breast cancer cohort.**

| **TILs distribution against subtypes w.r.t clinical parameters** |  | **Total (Mean ± S.E)** | ***p-values***  ***(Mann-Whitney test)*** |
| --- | --- | --- | --- |
| **No. Of patients (n=229)** | (Mean ± S.E) | 21.6 ± 1.4 (n=229) |  |
| **Age, (n=227)** | Early (<50) | 23.8 ± 2.4 (n=86) | 0.0856 |
|  | Late (≥ 50) | 20.2 ± 1.8 (n=141) |  |
|  | NA | 2 |  |
| **Menopausal status, (n=192)** | pre | 22.7 ± 3.0 (n=58) | 0.5474 |
|  | post | 21.5 ± 1.9 (n=134) |  |
|  | NA | 37 |  |
| **Grade, (n=229)** | I/II | 10.4 ± 0.9 (n=111) | **< 0.0001** |
|  | III | 32.1 ± 2.2 (n=118) |  |
|  | NA | 0 |  |
| **Tumor size (cT), (n=216)** | T1 | 15.4 ± 2.2 (n=66) | **0.0148**^$^ |
|  | T2 | 24.6 ± 2.0 (n=136) |  |
|  | T3,T4 | 17.9 ± 3.6 (n=14) |  |
|  | NA | 13 |  |
| **LN status(cN), (n=206)** | negative | 19.1 ± 2.6 (n=65) | 0.1052 |
|  | positive | 22.3 ± 1.8 (n=141) |  |
|  | NA | 23 |  |
| **Clinical Stage, (n=209)** | Early (<IIB) | 20.0 ± 2.5 (n=84) | 0.0969 |
|  | Late (≥IIB) | 22.7 ± 1.9 (n=125) |  |
|  | NA | 20 |  |
| **LVI*****, (n=229)** | Negative | 21.7 ± 1.6 (n=184) | 0.4723 |
|  | Positive | 20.9 ± 3.4 (n=45) |  |
|  | NA | 0 |  |
| **Tumor size (pT), (n=128) NACT_No** | T0, Tis | 32.5 ± 12.4 (n=6) | 0.4358^$^ |
|  | T1, T2 | 20.9 ± 2.0 (n=115) |  |
|  | T3,T4 | 19.3 ± 5.2 (n=7) |  |
|  | NA/NACT_Yes | 101 |  |
| **LN status(pN), (n=128) NACT_No** | Negative | 21.7 ± 2.4 (n=86) | 0.9918 |
|  | Positive | 20.8 ± 3.1 (n=42) |  |
|  | NA/NACT_Yes | 101 |  |
| **Pathological Stage, (n=128) NACT_No** | Early (<IIB) | 22.0 ± 2.5 (n=85) | 0.8129 |
|  | Late (≥IIB) | 20.1 ± 3.1 (n=43) |  |
|  | NA/NACT_Yes | 101 |  |

Mean±S.E (number of patients) of sTILs scores across clinicopathological parameters including age at diagnosis, menopausal status, tumour grade, radiological and pathological tumor size, lymph node positivity, stage, and LVI. For patients who did not receive NACT/NAHT, pT and pN retrieved from the surgery pathology report are noted. For comparing mean sTILs score differences across each parameter Mann-Whitney test was performed on GraphPad Prism v.5. The bold font indicates significant p-values. * LVI- lympho-vascular invasion ^$^ Kruskal Wallis test was performed for comparing mean sTILs scores for cT and pT.

**Figure S1: sTILs score distribution across clinicopathological parameters in IDC cohort and TNBC subtype.**

| 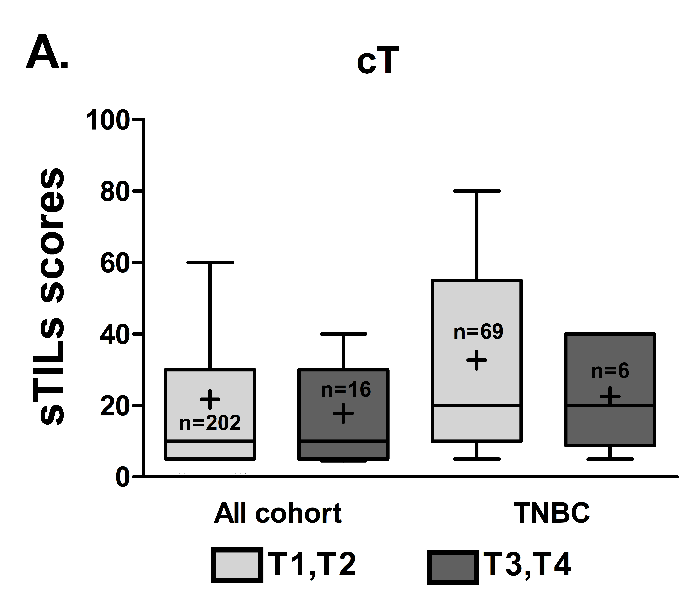 | 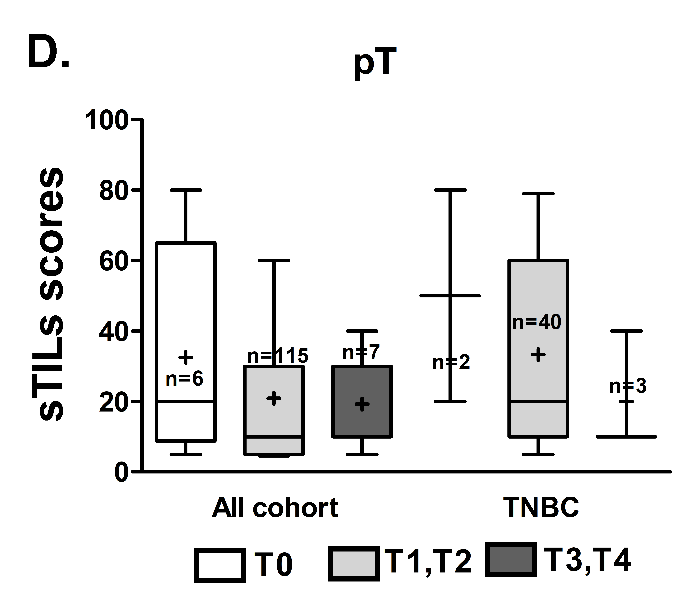 |
| --- | --- |
| 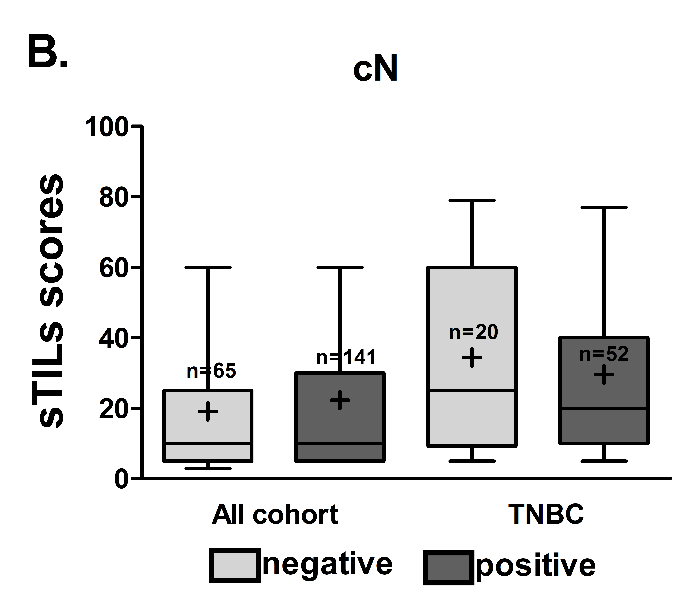 | 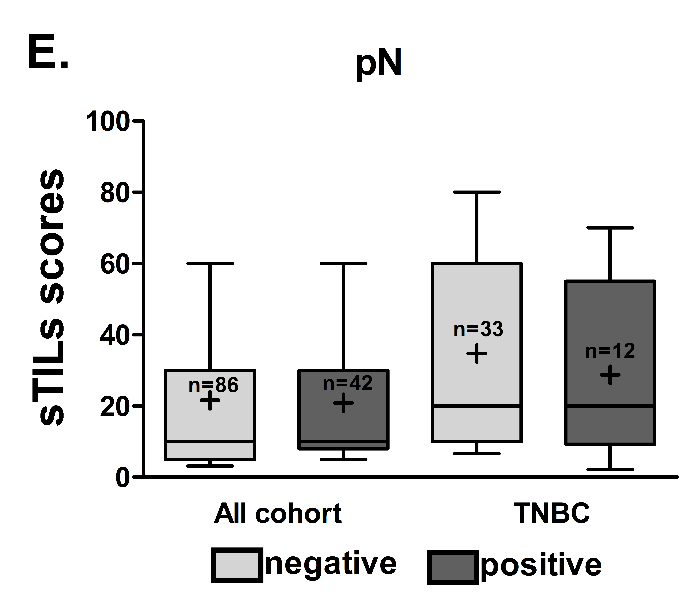 |
| 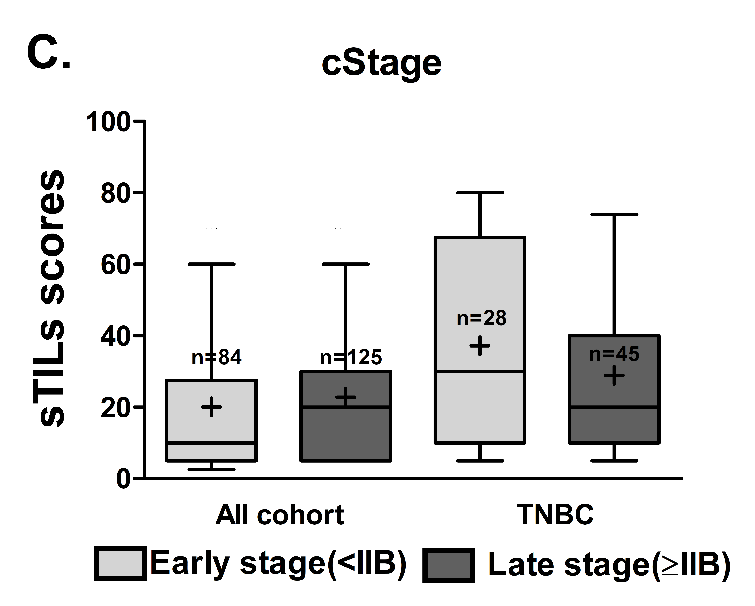 | 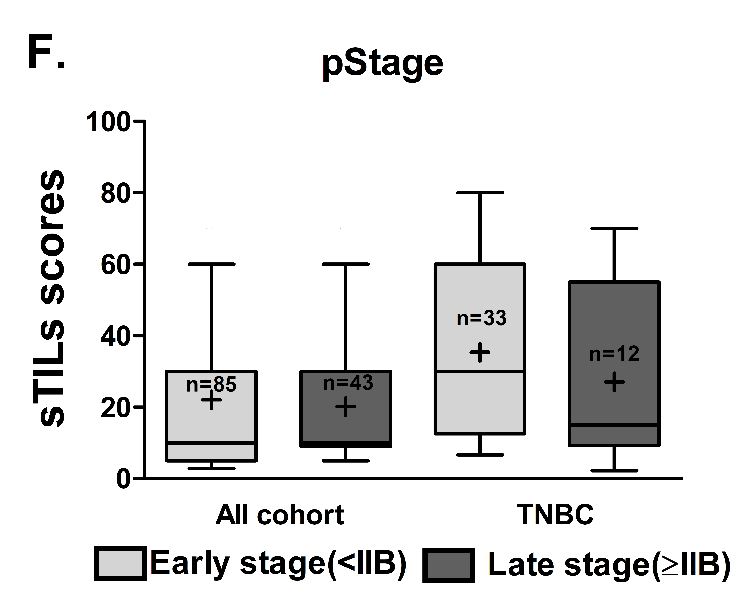 |

Box plots show sTILs scores for All breast cancer and TNBC cohort across; A. clinical tumor size, B. clinical lymph-node status, C. clinical-stage, D. pathological tumor size, E. pathological lymph-node status, F. pathological stage. The number of tissue samples (n) is shown on top of each bar. Error bars represent 10^th^ and 90^th^ percentile values. Kruskal-Wallis was performed to test significance across mean sTILs difference across all parameters. GraphPad Prism v.5 was used for the graphs and statistical calculations.

**Figure S2: sTILs mean scores between primary and post-NACT tumor samples with respect to clinicopathological parameters in IDC cohort and TNBC subtype.**

| 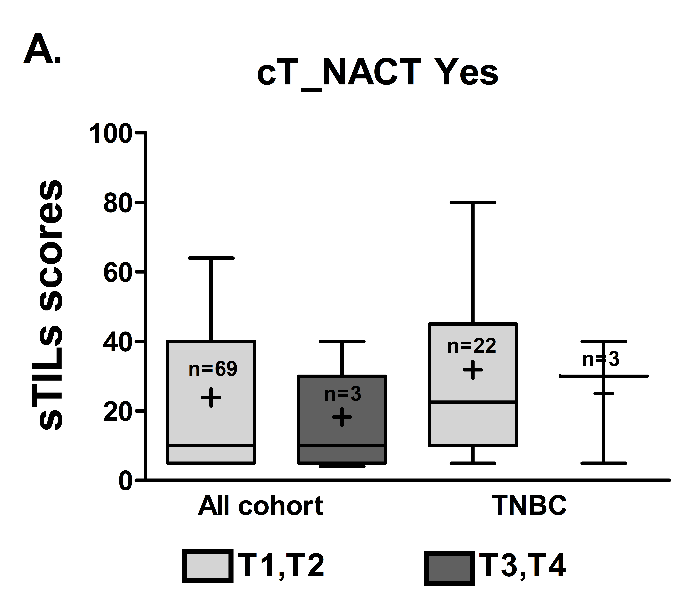 | 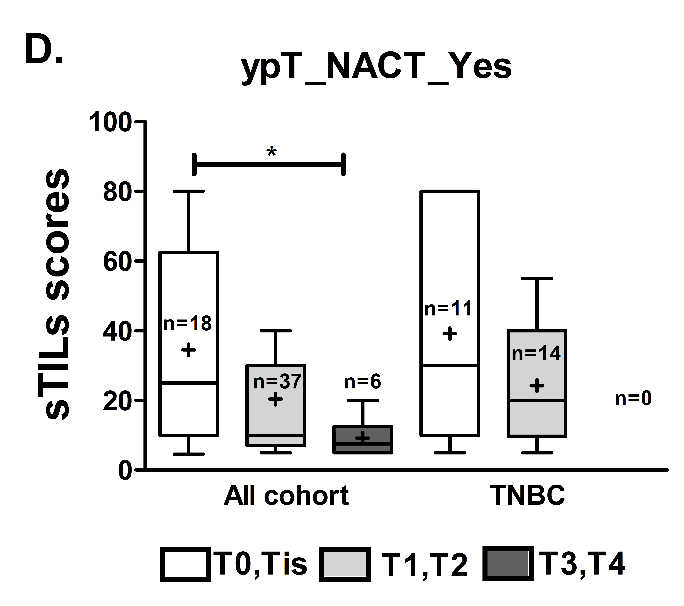 |
| --- | --- |
| 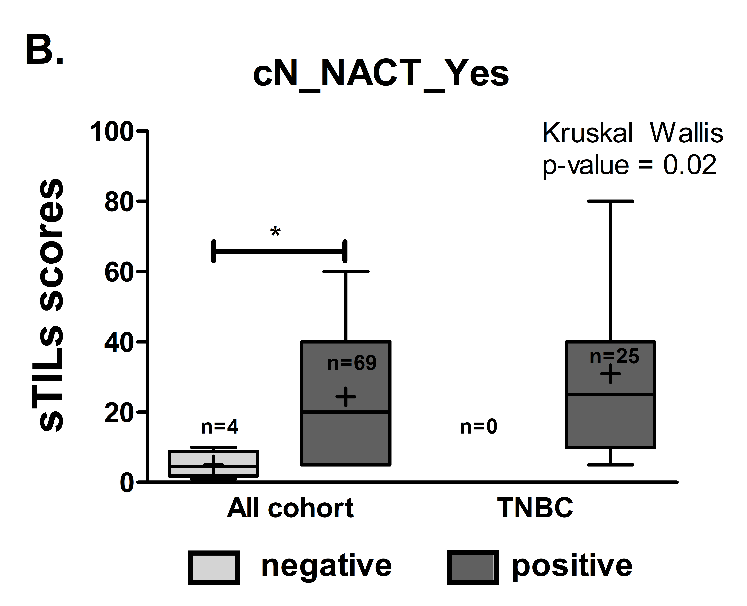 | 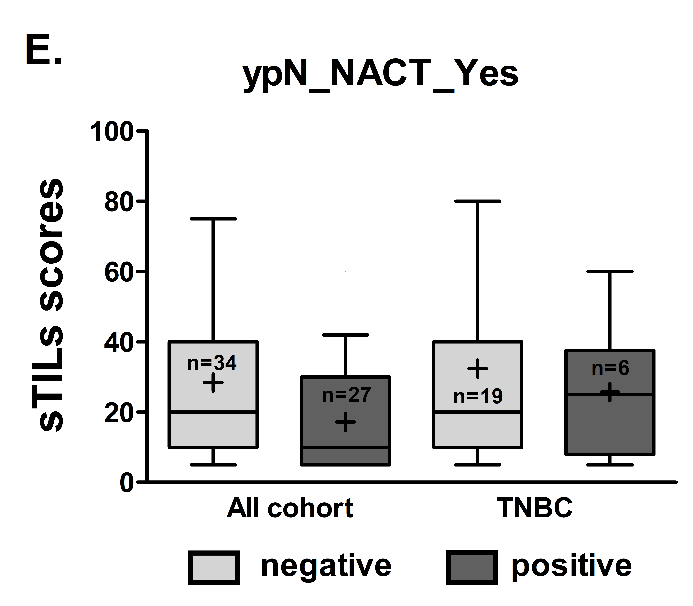 |
| 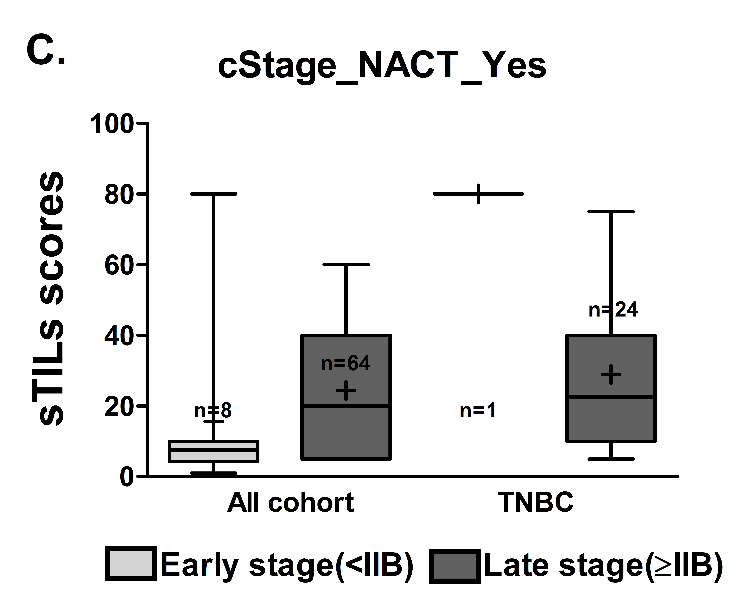 | 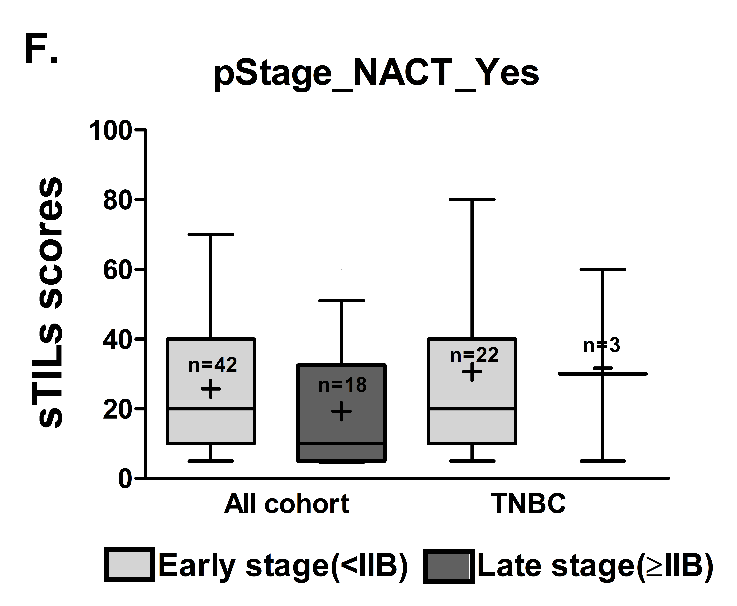 |

Box plots depicting sTILs scores for NACT treated patients (NACT_Yes) in All breast cancer cohort and TNBC subtype across A. clinical tumor size, B. clinical lymph-node status, C. clinical-stage, D. pathological tumor size, E. pathological lymph-node status, F. pathological stage. The number of tissue samples (n) is shown on top of each bar. Error bars represent 10^th^ and 90^th^ percentile values. Kruskal-Wallis was performed to test significance across mean sTILs difference across all parameters. Mann Whitney test was performed to test for significant sTILs scores difference between two parameters.

p-value <0.05 is represented with ‘*’. GraphPad Prism v.5 was used for the graphs and statistical calculations.

**Figure S3: Overall survival (DFS) for five-year follow-up according to the spatial TILs phenotype and sTILs scores.**


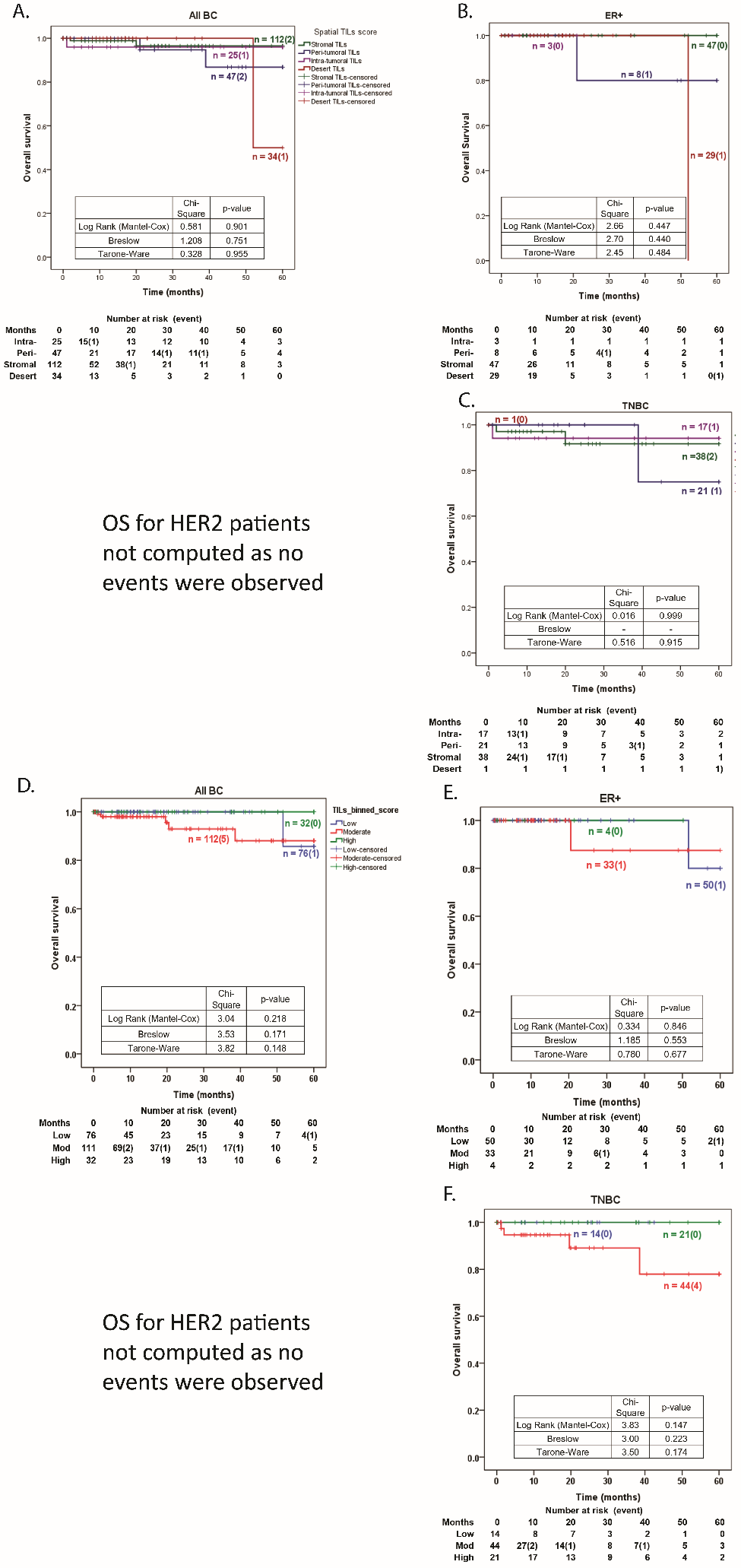


Overall survival (OS) was calculated as number of months from the date of biopsy till the last follow-up date up to five years. Kaplan-Meier survival plots for overall survival (OS) for 218 patients and 219 patients for spatial TILs phenotype and sTILs scores are plotted. Each drop shown as vertical line represents an event i.e., death due to disease. Survival probability with respect to spatial TILs scores is tested using IBM SPSS Statistics v. 21.0.0.0. Number of patients at risk at each time interval of 10months from 0-60 months is shown. The number of events is indicated in brackets at respective time points.

A-C: OS for four phenotypes of spatial TILs; Intra-tumoral TILs, Peri-tumoral TILs, Stromal TILs and Desert TILs for A. the IDC cohort, B. ER+ subtype and C. TNBC subtype. In the graph, X-axis represents the time scale in months, and Y-axis represents the survival probability. The green line indicates patients with stromal TILs, the purple line indicates patients with intra-tumoral TILs, and the red line indicates patients with desert TILs phenotype.

D-F: OS with respect to binned percent stromal TILs infiltration score. Kaplan-Meier survival plots for disease-free survival (DFS) according to low, moderate & high sTILs score bins for E; the IDC cohort, F; ER+ subtype, G; HER2+ subtype and H; TNBC. In the graph, X-axis represents the time scale in months, and Y-axis represents the survival probability. The blue line indicates patients with low sTILs scores, the red line indicates patients with moderate scores & green indicates high sTILs scores.
